# REM sleep behavior disorder: update on diagnosis and management

**DOI:** 10.1055/s-0043-1777111

**Published:** 2023-12-29

**Authors:** Manoel Alves Sobreira-Neto, Fernando Gustavo Stelzer, Lívia Leite Góes Gitaí, Rosana Cardoso Alves, Alan Luiz Eckeli, Carlos H. Schenck

**Affiliations:** 1 Universidade Federal do Ceará, Faculty of Medicine, Division of Neurology, Fortaleza CE, Brazil.; 2 Univeridade de São Paulo, Ribeirão Preto Medical School, Department of Neurosciences and Behavioral Sciences, Ribeirão Preto SP, Brazil.; 3 Universidade Federal de Alagoas, Faculty of Medicine, Division of Neurology, Maceió AL, Brazil.; 4 Fleury Medicine Group, Sleep Medicine Center, São Paulo SP, Brazil.; 5 Minnesota Regional Sleep Disorders Center; and University of Minnesota, Medical School, Departments of Psychiatry; and Hennepin County Medical Center, Minneapolis MN, United States of America.

**Keywords:** REM Sleep Behavior Disorder, Sleep, REM, Parasomnias, Synucleinopathies, Sleep Wake Disorders, Polysomnography, Drug Therapy, Transtorno do Comportamento do Sono REM, Sono REM, Parassonias, Sinucleinopatias, Transtornos do Sono-Vigília, Polissonografia, Tratamento Farmacológico

## Abstract

REM sleep behavior disorder (RBD) is characterized by a loss of atonia of skeletal muscles during REM sleep, associated with acting out behaviors during dreams. Knowledge of this pathology is important to predict neurodegenerative diseases since there is a strong association of RBD with diseases caused by the deposition of alpha-synuclein in neurons (synucleinopathies), such as Parkinson's disease (PD), multiple system atrophy (MSA), and dementia with Lewy bodies (DLB). Proper diagnosis of this condition will enable the use of future neuroprotective strategies before motor and cognitive symptoms. Diagnostic assessment should begin with a detailed clinical history with the patient and bed partner or roommate and the examination of any recorded home videos. Polysomnography (PSG) is necessary to verify the loss of sleep atonia and, when documented, the behaviors during sleep. Technical recommendations for PSG acquisition and analysis are defined in the AASM Manual for the scoring of sleep and associated events, and the PSG report should describe the percentage of REM sleep epochs that meet the criteria for RWA (REM without atonia) to better distinguish patients with and without RBD. Additionally, PSG helps rule out conditions that may mimic RBD, such as obstructive sleep apnea, non-REM sleep parasomnias, nocturnal epileptic seizures, periodic limb movements, and psychiatric disorders. Treatment of RBD involves guidance on protecting the environment and avoiding injuries to the patient and bed partner/roommate. Use of medications are also reviewed in the article. The development of neuroprotective medications will be crucial for future RBD therapy.

## Resumo

O transtorno comportamental do sono REM (TCSREM) é caracterizado por uma perda de atonia dos músculos esqueléticos durante o sono REM, associada a comportamentos de atuação durante os sonhos. O conhecimento desse transtorno é importante como preditor de doenças neurodegenerativas, uma vez que existe uma forte associação de TCSREM com doenças causadas pela deposição de alfa-sinucleína nos neurônios, como a doença de Parkinson (DP), atrofia de múltiplos sistemas (MSA) e demência com corpos de Lewy (DLB). O diagnóstico adequado dessa condição permitirá o uso de futuras estratégias neuroprotetoras antes do aparecimento dos sintomas motores e cognitivos. A avaliação diagnóstica deve começar com uma história clínica detalhada com o paciente e acompanhante, além de exame de vídeos. A polissonografia (PSG) é necessária para verificar a perda da atonia do sono e, quando documentados, os comportamentos durante o sono. As recomendações técnicas para aquisição e análise de PSG são definidas no Manual da AASM ( *Scoring of sleep and associated events* ) e o relatório de PSG deve descrever a porcentagem de períodos de sono REM que atendem aos critérios para REM sem atonia. Além disso, a PSG ajuda a descartar condições que podem mimetizar o TCSREM, como apneia obstrutiva do sono, parassonias do sono não REM, crises epilépticas noturnas, movimentos periódicos dos membros e transtornos psiquiátricos. O tratamento do TCSREM envolve orientações sobre adaptações do ambiente para evitar lesões ao paciente e ao colega de quarto. Medicamentos utilizados são revistos no artigo, assim como o crucial desenvolvimento de medicamentos neuroprotetores.

## INTRODUCTION

REM Sleep Behavior Disorder (RBD) is characterized by the loss of atonia of skeletal muscles during REM sleep, associated with acting out behaviors during dreams and/or nightmares. 1 Knowledge of this pathology is crucial to avoid harm to the patient and their bed partner, as well as to predict, when occurring in isolated form in middle-aged and older adults, neurodegenerative diseases, such as synucleinopathies. Therefore, the proper diagnosis of the condition will help enroll these patients in future neuroprotective trials before the motor and cognitive symptoms become clinically manifest.

**Figure 1:**
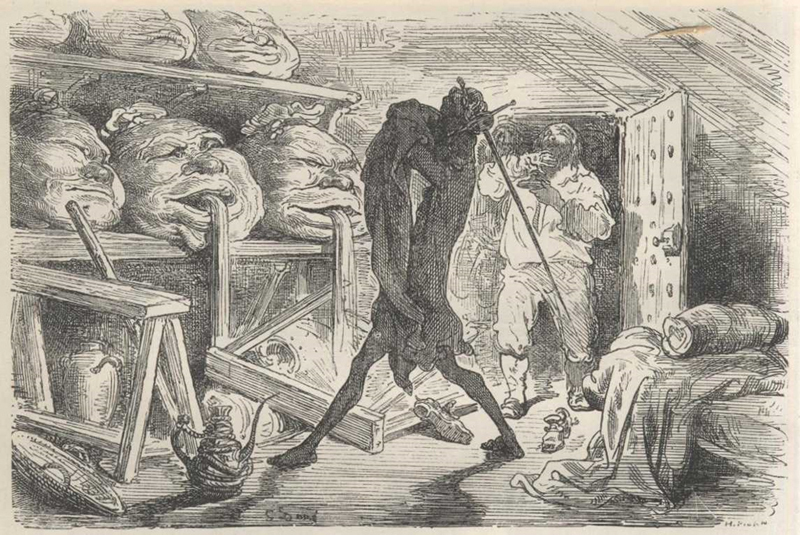
Probable REM Sleep Behavior Disorder episode in Miguel Cervantes Don Quixote de La Mancha: they found Don Quixote in the strangest costume in the world. (…) on his head, he had a little greasy red cap (…), and in his right hand he held his unsheathed sword, with which he was slashing about on all sides, uttering exclamations as if he were fighting some giant: and the best of it was his eyes were not open, for he was fast asleep, and dreaming that he was doing battle with the giant. For his imagination was so wrought upon by the adventure he was going to accomplish, that it made him dream he had already reached the kingdom of Micomicon and was engaged in combat with his enemy; and believing he was slaying the giant, he had given so many sword cuts to the (wine) skins that the whole room was full of wine. (…) despite all this the poor gentleman never woke up until the barber brought a great pot of cold water from the well and flung it with one dash all over his body, on which Don Quixote woke up, but not so completely as to understand what was the matter. Source: Gustave Doré, in: Illustrations for Don Quixote de la Mancha, 1863. Accessed in https://pt.wikipedia.org/wiki/Dom_Quixote_(Gustave_Dor%C3%A9 ) - public domain image.

The first description of REM sleep without atonia (RWA) and dream-related behaviors was made by Jouvet (1965) in cats with lesions in the subcoeruleus nucleus region. In the 1970s, Japanese authors described this condition in patients during alcohol withdrawal and called it Stage I-REM with tonic electromyography. In 1986, Schenck and colleagues described a series of patients who exhibited dream-enacting behaviors and loss of atonia during REM sleep, coining for the first time the term RBD 2 3 Preceding RBD scientific descriptions, several artistic works portrayed probable episodes compatible with this diagnosis. 3 In Don Quixote de la Mancha (1605 - 1615), Miguel de Cervantes describes several sleep disorders, including a probable episode of RBD 4 ( Figure 1 ).

## PHYSIOPATHOLOGY

In the study of REM sleep physiology, there are two cellular groups (“REM on” and “REM off” cells) that play a role in the initiation and maintenance of REM sleep through reciprocal inhibition between these groups (Mesopontine “Flip-Flop” model). 5 The “REM on” cells consist of the precoeruleus nucleus (glutamatergic) and sublaterodorsal nucleus (SLD) (glutamatergic and GABAergic), located in the caudal tegmentum of the pontis. On the other hand, the “REM off” cells are formed by the ventrolateral part of the periaqueductal gray and the pontine ventrolateral tegmentum (both GABAergic), located in the rostral tegmentum of the pons. 6

These two “REM on” cell nuclei probably have different functions. The precoeruleus nucleus likely regulates electroencephalographic activity, while the SLD nucleus is responsible for muscular atonia and the inhibition of “REM off” cells. These cellular groups are heavily modulated by cholinergic neurons (pedunculopontine nucleus and laterodorsal tegmental nucleus), which are activated during REM sleep, and by monoaminergic cells (locus coeruleus and raphe nucleus), which are inhibited during REM sleep. 7 8

**Figure 2:**
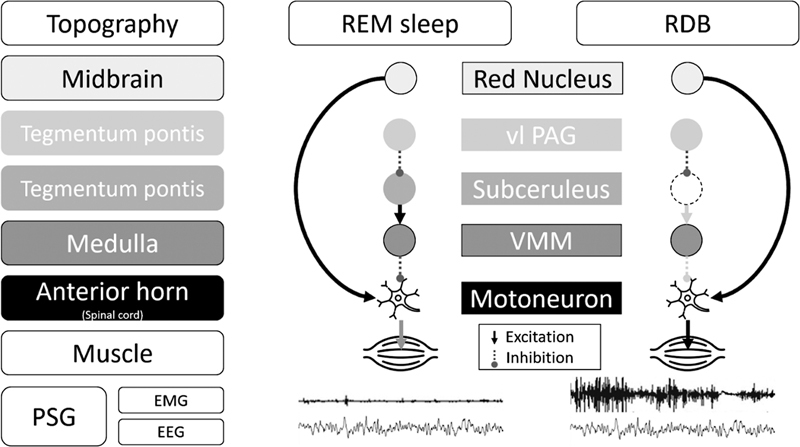
Abbreviations: EEG, electroencephalography; EMG, electromyography; PSG, polysomnography; vl PAG, ventrolateral periaqueductal gray matter; VMM, ventral medial medulla. Adapted from Fraigne et al., 2015. 9 Representation of the regulation of muscle activity during physiologic REM sleep and REM sleep behavior disorder (RBD). In physiologic conditions, glutamatergic subcoeruleus neurons regulate REM sleep muscle tonus through the excitation of inhibitory neurons of the ventromedial medulla (VMM). Those neurons inhibit motoneurons in the anterior horn of the spinal cord. In REM sleep, physiological transient muscle activity can be observed, mediated by red nucleus activity. In individuals with RBD, degeneration of the circuit subcoeruleus-ventromedial medulla releases motoneurons from their physiologic inhibition, allowing motor behaviors during REM sleep.

Two different systems mediate motor control during REM sleep. One is responsible for locomotor drive and is formed by locomotion generators located in the brainstem, which receive influences from the telencephalon and diencephalon. The other system is responsible for inhibiting muscle tone in the anterior horn of the spinal cord. It is constituted by the aforementioned “REM on” cells, primarily the magnocellular reticular formation located in the medulla oblongata. 7 RBD appears to result from a combination of increased locomotor drive associated with lesions in nuclei responsible for atonia during REM sleep (magnocellular reticular formation and sublaterodorsal nucleus). Therefore, it involves a complex and multifaceted neurotransmitter dysfunction 7 9 ( Figure 2 ).

## EPIDEMIOLOGY

Since diagnostic methods for RBD are not widely available in many centers, epidemiological data regarding its prevalence is limited. Epidemiological studies based on clinical interviews and questionnaires, without polysomnographic confirmation, estimated a prevalence ranging from 3–10%. Prevalence estimated from community-based studies, with RDB diagnosis based on clinical and polysomnographic data, estimated a prevalence between 0.74 to 1.15%. 10

Additionally, in regard to alpha-synucleinopathies, the main RBD risk factor involves age over 50 years old, and perhaps also male gender, although a Swiss study found an equal male-female RBD ratio in a community-based PSG study of middle-aged and older adults, as discussed. 10 In individuals younger than 50 years old, there is a similar prevalence of RBD in men and women. In this specific population (those under 50 years old), narcolepsy type 1 and medication use are important association factors. 10

## NEUROIMAGING

Neuroimaging studies using positron emission tomography (PET), single-photon emission computed tomography (SPECT), and magnetic resonance imaging (MRI) modalities are contributing to a better neurochemical, structural, and functional understanding of RBD.

Regarding molecular changes, patients with isolated RBD showed intermediate dopamine transporters (DAT) levels in the striatum, lower than healthy controls but higher than PD and RBD patients. 11 A deficit of dopaminergic markers in patients with isolated RBD was associated with a greater likelihood of developing an alpha-synucleinopathy over 3 to 5 years of follow-up. 12 Cortical levels of acetylcholinesterase were reduced in patients with isolated RBD compared to controls. 13 PD patients with RBD also exhibited a greater loss than PD patients without RBD. The literature on regional cerebral blood flow was contradictory but suggested abnormal perfusion throughout the brain in patients with isolated RBD compared to healthy controls. Glucose metabolism was also dysregulated in patients with isolated RBD, with hypometabolism in the posterior parts of the brain and hypermetabolism in other regions. 14

As for the structural assessment, MRI literature revealed that abnormal iron deposits can be found in subcortical nuclei of the brainstem, a pathophysiological hallmark of iRBD, namely the substantia nigra and locus coeruleus. 15 Widespread cortical atrophy occurs in iRBD patients, showing distinct anterior (involving orbitofrontal cortex) and posterior (including parietal, occipital, and temporal cortices) patterns. We understand that a continuum links iRBD to more pronounced brain abnormalities in PD. In PD with RBD, atrophy is observed in limbic and basal ganglia regions compared to PD without RBD and healthy controls, consistent with iRBD pathology.

The functional studies identified cortico-cortical, nigrostriatal, and striato-cortical functional connectivity disruptions in iRBD patients correlated with cognitive decline, autonomic dysregulation, and motor impairment. 16 Compensatory mechanisms were observed with varying cognitive loads during motor tasks, indicating heightened recruitment of functional networks even with low cognitive demands. 17 Frontal connectivity pathways involving somatosensory regions were noted for maintaining motor function. Like structural MRI findings, a cortical posterior-based functional connectivity signature, frontostriatal deficits, and insular connectivity dysfunction were observed in iRBD patients, mirroring those in PD. 18 Consistent patterns were also seen in PD patients with RBD, with disruptions in cortico-cortical and striato-cortical functioning.

## CLINICAL MANIFESTATIONS AND DIAGNOSIS

Motor behaviors and vocalizations during REM sleep, associated with dreams and nightmares, characterize RBD. Such behaviors can cause injuries to the patient and their partners, commonly observed as pushing, kicking, punching, biting, shouting, cursing, and occasionally more dangerous acts such as choking. 19 20 Some studies report that patient or partner injuries occur in 48 to 77% of cases. 7

At the end of the episodes, the individual may wake up rapidly, and when asked about their dreams, they report a story consistent with the behaviors exhibited during sleep. This phenomenon is known as isomorphism. 7 The dreams are often negative, with reports of being attacked, pursued, or assaulted by strangers or animals and falling from cliffs. 20 21 During the episodes, it is not common for individuals to walk, run, or leave the room. Additionally, patients typically keep their eyes closed during the events. These characteristics aid in differentiating RBD from other parasomnias and nocturnal epileptic seizures. 22

Although less frequent, non-violent behaviors may also occur in these patients, who often consider it normal. 20 21 Descriptions of these phenomena are wide-ranging. Patients behave as if they were smoking, giving speeches to an audience, or flying. Vocalizations such as talking, screaming, crying, laughing, singing, and whispering occur frequently. Other behaviors like eating and sexual activities could also happen. 23 In some patients, a coexistence of violent and non-violent behaviors is present. 20 21

These behaviors usually begin after 90 minutes of sleep onset, mainly in the second half of the night when REM sleep is more prevalent. The presence of a witness who can report abnormal behaviors during sleep is essential since some patients do not recall the content of their dreams and movements during sleep. 22 The RBD behaviors vary from night to night and over time in the same individual and between patients. Small twitches and brief jerks affecting the extremities are the most common movements in RBD and may remain unnoticed for years.

**Figure 3:**
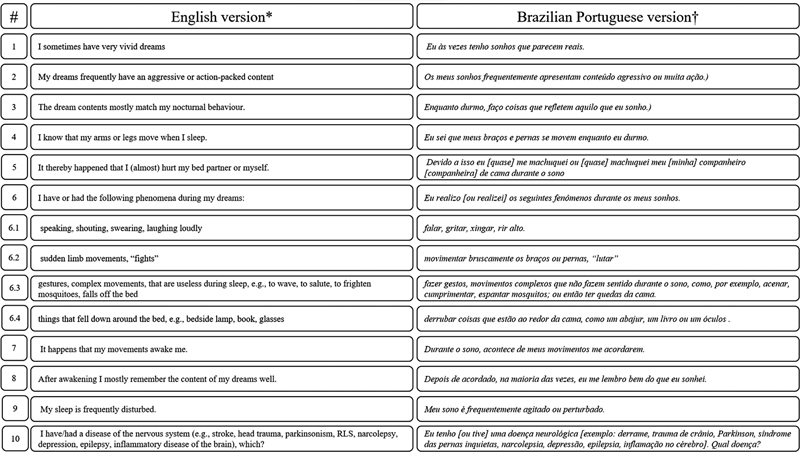
REM Sleep Behavior Disorder (RDB) Screening Questionnaire - English version (*) and Brazilian Portuguese version (†). Source: Stiasny-Kolster et al., 2007; Pena-Pereira et al., 2020. 24 25

Screening instruments, such as scales, can be utilized for screening patients. The REM Sleep Behavior Disorder Screening Questionnaire (RBDSQ) 24 has been translated and validated for Brazilian Portuguese. 25 It is a self-administered instrument with ten simple questions, scored from 0 to 13. Cut-off values of more than four have shown good sensitivity for the suspicion of RBD ( Figure 3 ).

The diagnostic evaluation should begin with a detailed clinical history with the patient and bed partner and the examination of recorded home videos, if available. After this initial stage, polysomnography (PSG) is necessary to identify and quantify RWA and to document sleep behaviors ( Table 1 ). Additionally, clinical evaluation helps to rule out conditions that may mimic RBD, such as Obstructive Sleep Apnea, non-REM sleep parasomnias, nocturnal epileptic seizures, sleep-related movement disorders, and psychiatric disorders (in particular Post-traumatic stress disorder). 1

**Table 1:** International Classification of Sleep Disorders 3 ^rd^ TR Diagnostic Criteria for REM Sleep Behavior Disorder. 1

CRITERIA A-D MUST BE MET
A. Repeated episodes of sleep-related vocalization and/or complex motor behaviors. B. These behaviors are documented by polysomnography to occur during REM sleep, or based on clinical history of dream enactment, are presumed to occur during REM sleep. C. Polysomnographic recording demonstrates RWA. D. The disturbance is not better explained by another sleep disorder, mental disorder, medication, or substance use.

Abbreviations: ICSD, International Classification of Sleep Disorders; RBD, REM sleep behavior disorder; RWA, REM sleep without atonia.

Notes: To evaluate amplitude, consider the lowest amplitude in NREM if no stage R atonia is present. If a periodic limb movement is scored as part of a PLMS series, it should not be counted in determining if an epoch has RWA.

Type 1 PSG is essential for RBD diagnosis. It is necessary to detect RWA, as required in the International Classification of Sleep Disorders, 3rd edition (ICSD-3). 1 Additionally, it allows documentation of complex motor behaviors and vocalizations during REM sleep. 1 SG can also aid in the differential diagnosis of conditions that may mimic or be comorbid with RBD, such as obstructive sleep apnea (OSA), non-REM parasomnia, sleep-related hypermotor epilepsy, vigorous periodic leg movements during sleep, trauma-associated sleep disorder and sleep-related movement disorder. 26

The PSG aimed at evaluating complex nocturnal behaviors should ideally include, in addition to the routine parameters, a series of additional variables. Therefore, it is necessary to use equipment with enough monitoring channels. Furthermore, these exams should always be done in the laboratory (type 1 PSG), with trained technicians who document and act during sleep movements.

The PSG analysis for diagnosing parasomnias involves a series of considerations related to the monitored parameters, careful analysis of muscle activity, and detailed examination of synchronized video. These make PSG analysis for diagnosing complex movements during sleep more sophisticated than routine sleep lab exams, used, for example, to diagnose sleep-related breathing disorders.

**Figure 4:**
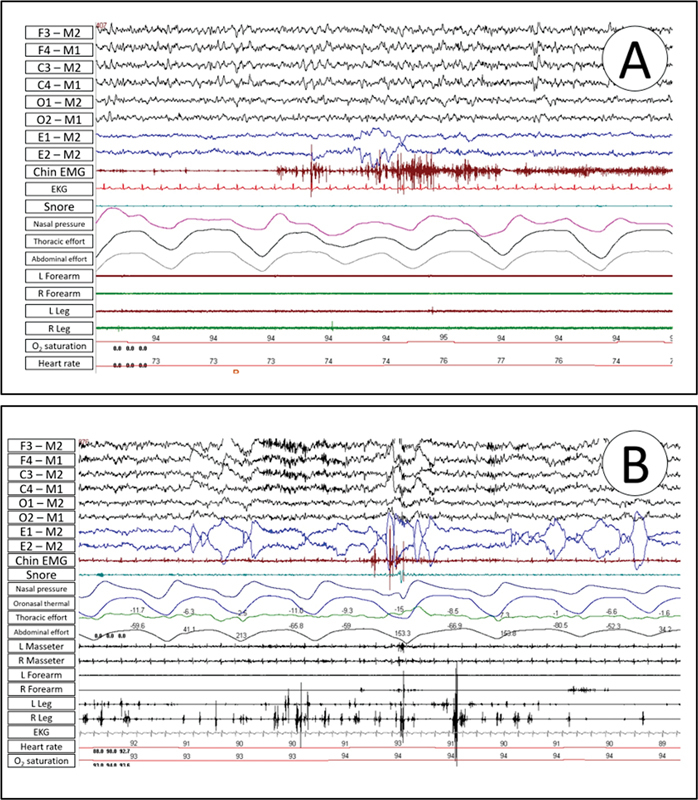
Abbreviations: EKG, electrocardiogram; EMG, electromyography; L, left; O2, oxygen; R, right. ( **A** ) REM sleep without atonia findings in polysomnography. It shows a 30 seconds epoch with excessive sustained tonic muscle activity observed in the chin electromyography derivation. ( **B** ) shows a 30 seconds epoch with excessive phasic muscle activity on the lower (anterior tibialis muscle) and upper (flexor digitorum superficialis muscle) limbs electromyography derivations.

The American Academy of Sleep Medicine Manual for the Scoring of Sleep and Associated Events defines technical recommendations for PSG acquisition and analysis. These recommendations likely pertain to the specific electrode placements, data collection procedures, and criteria for scoring various sleep stages and events during the PSG ( Table 2 ) ( Figure 4 ). 1

**Table 2:** American Academy of Sleep Medicine specifications for detection of REM Sleep without atonia. 30

TECHNICAL SPECIFICATIONS FOR MONITORING
1. Time-synchronized, audio-equipped video PSG should be used to document complex motor behaviors and vocalizations during REM sleep. 2. LMs - surface electrodes placed longitudinally and symmetrically in the middle of the anterior tibialis muscle so that they are 2-3 cm apart or one-third of the length of the anterior tibialis muscle, whichever is shorter. 3. For monitoring LMs, the use of 60 Hz (notch) filters should be avoided. Impedances need to be < 10,000 Ω. 5,000 Ω is preferred but may be difficult to obtain. 4. For monitoring movements of the upper limbs, surface electrodes should be placed longitudinally and symmetrically, so that they are 2-3 cm apart over the surface of the flexor digitorum superficialis or the surface of the extensor digitorum communis. 5. Both legs and arms should be monitored. Separate channels for each leg and each arm are strongly preferred.
**DEFINITIONS**
1. **Excessive sustained muscle activity (tonic activity) in REM:** An epoch of stage R with at least 50% of the duration of the epoch having a chin EMG amplitude at least 2 times greater than the stage R atonia level (or lowest amplitude in NREM, if no stage R atonia is present). Multiple segments may contribute to the total duration, but each segment must be > 5 seconds. 2. **Excessive transient muscle activity (phasic activity) in REM:** In a 30-second epoch of stage R divided into 10 sequential 3-second mini-epochs, at least 5 (50%) of the mini-epochs contain bursts of transient muscle activity in the chin or limb EMG. In RWA, excessive transient muscle activity bursts are 0.1-5.0 seconds in duration and at least 2 times as high in amplitude as the stage R atonia level. 3. **Any chin EMG activity** . Activity with a minimum amplitude 2 times greater than the stage R atonia level.
**CRITERIA**
**AN EPOCH EXHIBITS RWA WHEN ONE OF THE FOLLOWING IS PRESENT:** a. Excessive sustained muscle activity in REM in the chin EMG. b. Excessive transient muscle activity during REM in the chin or limb EMG. c. At least 50% of 3-second mini-epochs contain any chin activity (as defined above) or limb EMG activity (bursts of EMG activity 0.1-5.0 seconds in duration and at least 2 times as high in amplitude as the stage R atonia level. **SCORE THE RWA INDEX AS THE PERCENT OF STAGE R EPOCHS THAT MEET CRITERIA**

Abbreviations: LMs, leg movements; PSG, polysomnography; RWA, REM sleep without atonia.

Notes: To evaluate amplitude, consider the lowest amplitude in NREM if no stage R atonia is present. If a periodic limb movement is scored as part of a PLMS series, it should not be counted in determining if an epoch has RWA. Epochs containing RWA with sustained chin activity as defined above may not meet criteria for stage R, but in these cases, the epoch can still be scored as stage R if other criteria for stage R are met, or if the epoch is contiguous with an epoch scored as stage R.

Since the AASM manual lacks a specific quantification of RWA to define clear cutoff values, other methods have been developed. 27 In the 'SINBAR' method, proposed by the Barcelona and Innsbruck group, the combination of 'any' EMG activity in the mentalis muscle with both phasic flexor digitorum superficialis muscles yielded a cutoff of 32% (area under the curve [AUC] 0.998) for patients with RBD. 28 The Mayo Clinic method combined visual analysis and a computer-based quantitative automatic scoring algorithm known as the 'REM sleep Atonia Index' to evaluate EMG activity in the submental and anterior tibialis muscles, yielding a cutoff of 43.4% (AUC 0.983). 29 In the same study, using the AASM diagnostic standards resulted in a combined submentalis and anterior tibialis phasic muscle activity cutoff of 34.7% (AUC 0.960). 29 Therefore, the polysomnography report should describe the percentage of REM sleep epochs that meet the criteria for RWA to better distinguish patients with and without RBD. 30

## RBD MIMICS

Some clinical conditions can mimic dream enactment behaviors, making it sometimes difficult to distinguish solely based on medical history or even homemade videos ( Table 3 ). OSA, especially in severe cases, can show upon awakening after a breathing event, particularly during REM sleep, with movements that align with performance during sleep. 31 Such behaviors tend to improve after treating the sleep-breathing disorder. Thus, snoring, feeling of choking, and witnessed apneas during sleep should draw attention to this differential diagnosis. Distinguishing between these conditions is crucial as clonazepam, one of the treatments used for RBD can worsen OSA.

Non-REM sleep parasomnias such as sleepwalking, night terrors, confusional arousal, and sleep-related eating disorders typically occur in younger individuals compared to RBD. Additionally, unlike RBD, where the patient exhibits behavior with closed eyes and in the final third of the night, in these parasomnias, the patient experiences symptoms in the early third of the night with eyes open. Since the occurrence during the polysomnography examination night is rare, the absence of REM sleep without atonia and multiple awakenings during slow wave sleep are hallmarks of non-REM sleep parasomnias. 26

Nocturnal epileptic seizures can also simulate RBD. Seizures originating in the frontal lobe can exhibit hyperkinetic motor manifestations, with abrupt, violent movements and intense limb activity, often confused with the aggressive behaviors of RBD. Furthermore, temporal lobe seizures can involve motor and verbal automatisms that may resemble the sleep talking observed in RBD. The stereotyped movements of epileptic seizures can help differentiate them, as their occurrence predominantly during non-REM sleep, the electroencephalographic changes such as epileptic discharges, and the response to anti-seizure medications. 32

Other less common clinical conditions can mimic RBD. Periodic limb movements during sleep (PLMS) are characterized by repetitive movements involving toe extension, ankle dorsiflexion, and sometimes knee flexion during sleep. Several cases of vigorous PLMS were mistakenly diagnosed as RBD during clinical evaluation. 33 Trauma-associated sleep disorder presents with trauma-related nightmares, autonomic hyper-arousal, and excessive movements, which can be confused with RBD-like symptoms. Another rare RBD mimic is Rhythmic Movement Disorders during sleep, involving repetitive, stereotyped, and rhythmic movements of large muscle groups. These movements occur during quiet wakefulness, drowsiness, or sleep. 26

## ASSOCIATED CONDITIONS

A strong association of RBD with diseases caused by the deposition of alpha-synuclein in neurons, such as Parkinson's disease (PD), Multiple System Atrophy (MSA), and Dementia with Lewy Bodies (DLB), is well-established. 34 35 36 The abnormal deposition of this protein in brainstem structures, particularly in the medulla oblongata and pontine transition, leads to the formation of intraneuronal inclusion bodies called Lewy bodies and the RBD clinical manifestations. 37 A prospective multicenter study involving 24 centers documented an annual phenoconversion rate of 6.3%, with a 73.5% phenoconversion rate after 12 years of follow-up. The majority of individuals developed PD (56.5%), followed by DLB (43.5%) and MSA (4.5%). 38

**Figure 5:**
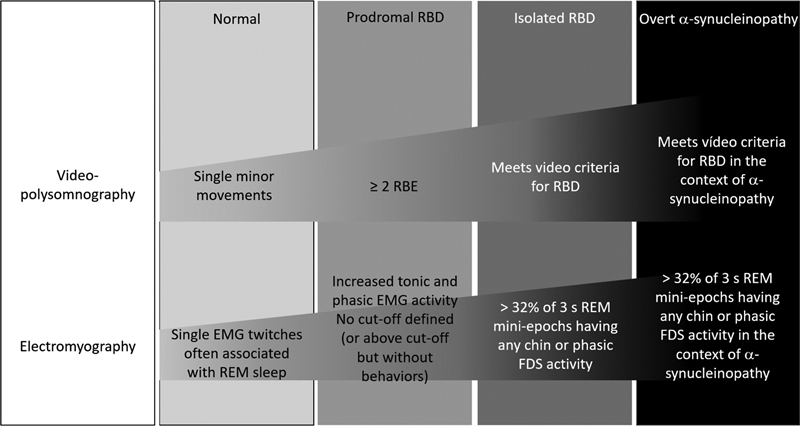
Abbreviations: FDS, flexor digitalis superficialis; RBE, REM sleep behavioral events. Concept of prodromal REM Sleep Behavior Disorder (RBD). Clinical and neurophysiological findings on type I video-polysomnography and electromyography (EMG) progress along a *continuum* over disease evolution. Individuals progress from isolated RBD into RBD in the context of overt alpha-synucleinopathy. Source: Högl et al, 2017. 3

In cases where no association with another condition exists, we refer to it as isolated RBD. There is an increasing body of evidence that suggests that most cases of isolated RBD are associated with asymptomatic alpha-synucleinopathies 3 39 40 ( Figure 5 ). Several studies have already demonstrated that patients with isolated RBD more frequently exhibit cognitive deficits, particularly in executive function, visuospatial attention, processing speed, short-term memory, and pareidolias (tendency to perceive a meaningful image in an ambiguous visual pattern). Almost a third of RBD patients met the diagnostic criteria for mild cognitive impairment (MCI), which serves as a predictive biomarker for DLB. 41 42 Other symptoms observed in RBD patients include hyposmia, constipation, orthostatic hypotension, and gait abnormalities, also linked to a higher risk of phenoconversion. 42

Besides the association with synucleinopathies, RBD may be related to medications, most frequently antidepressants, and substance withdrawal. Other neurologic conditions were associated with RBD, such as narcolepsy type 1. RBD can also manifest as a consequence of a neurological lesion affecting structures related to REM sleep atonia control, in the brainstem. Such conditions are relatively rare, but secondary RDB can occur in the context of meningiomas or, even more rarely, pontine cavernomas or lymphomas, multiple sclerosis, and stroke. RBD can also be associated with posttraumatic stress disorder 43 44 ( Table 4 ).

**Table 3:** Main clinical differential diagnosis of REM Sleep Behavior Disorder.

**Sleep disorders** Obstructive sleep apnea Non-REM parasomnias • Sleepwalking • Sleep terrors • Confusional arousals, with aggressive/violent behaviors Parasomnia overlap disorder (RBD + Non-REM parasomnia) Periodic leg movements during sleep Sleep Related rhythmic movement disorder Status dissociatus (aggressive/violent behavior during ambiguous sleep)
**Neurological disorders** Nocturnal epileptic seizures o Sleep-related hypermotor epilepsy Anti-IgLON5 disease
**Psychiatric disorders** Post-traumatic stress disorder Sleep-related dissociative disorder
**Other clinical disorders** Nocturnal (severe) hypoglicemia (insulinoma)

Sources: Antelmi E, et al. 2021 and Baltzan M, et al., 2020. 26 43

**Table 4:** Conditions and substances associated with REM Sleep Behavior Disorder and REM sleep without atonia.

**Conditions**
**1) Alpha-synucleinopathy:** ○ Dementia with Lewy bodies (prevalence of RBD: 68–90%) ○ Parkinson's disease (prevalence of RBD: 15–65%) ○ Multiple system atrophy (prevalence of RBD: 60–90%) **2) Sleep disorders:** ○ Narcolepsy type 1 (prevalence of RBD: up to 60%) 3) Paraneoplastic and autoimmune encephalitis: ○ Voltage-gated potassium channel complex (contactin-associated protein 2 [CASPR2], leucine-rich glioma-inactivated protein 1 [LGI1]) ○ Ma1 and Ma2 autoantibody-related encephalopathies· Anti-IgLON5 disease **4) Other neurologic diseases:** ○ Machado-Joseph disease (spinocerebellar atrophy type 3) ○ Adult-onset autosomal dominant leukodystrophy ○ Myotonic dystrophy type 2 ○ Autism ○ Gilles de la Tourette syndrome ○ Möbius syndrome ○ Smith-Magenis syndrome ○ Amyotrophic lateral sclerosis ○ Progressive supranuclear palsy ○ Huntington's disease ○ Wilson's disease ○ Creutzfeldt-Jakob disease ○ Multiple sclerosis ○ Astrocytomas ○ Acoustic neuroma ○ Vascular malformations ○ Central nervous system vasculitis ○ Cerebral infarcts ○ Guadeloupean atypical parkinsonism
**Medications**
**1) Antidepressants** **1.a. Selective serotonin reuptake inhibitor:** ○ Citalopram ○ Fluoxetine ○ Paroxetine ○ Sertraline **1.b. Serotonin-norepinephrine reuptake inhibitor:** ○ Duloxetine ○ Venlafaxine ○ Mirtazapine* * (atypical mechanism of action) **1.c. Selective norepinephrine reuptake inhibitor:** o Reboxetine **1.c. Tricyclic antidepressant:** ○ Amitriptyline ○ Clomipramine ○ Imipramine ○ Nortriptyline 2) Lithium 3) Monoamine oxidase inhibitor: ○ Phenelzine ○ Selegiline **4) Beta blocker:** ○ Bisoprolol ○ Propranolol 5) Caffeine abuse 6) Withdrawal from substances: ○ Barbiturates ○ Benzodiazepines (nitrazepam) ○ Ethanol ○ Amphetamines ○ Cocaine

Adapted from Hoque & Chesson, 2010; Baltzan et al., 2020; AASM, 2023.

## TREATMENT

The treatment of RBD involves safety orientation to protect the environment and avoid injuries to the patient and the sleep partner. Sharp and puncture-prone objects, weapons, and any objects on the nightstand that could lead to safety issues should be removed from the bedroom. Additional safety measures include safety bars and nets on windows and balconies, padded corners and edges of furniture, placement of the mattress on the floor, or use of side safety rails to prevent falls from the bed. Protecting the sleep partner requires protective barriers, such as the placement of pillows between the patient and the sleep partner or else sleeping in separate beds with a safe distance between them. 45

In individuals with drug-induced or drug-exacerbated RBD, drug discontinuation is only recommended if clinically safe for the patient, particularly in the context of psychiatric disorders when the psychiatrist should be consulted. For example, dream enactment may improve after decreasing or discontinuing an antidepressant, but clinicians should remember that this may take several months. If antidepressant discontinuation is not psychiatrically safe, bupropion, a drug with a lower serotoninergic profile, may be an option, especially since no case of bupropion-induced or exacerbated RBD has been reported. 45

Until now, few double-blind randomized trials have evaluated medication use for RBD. Most of the published papers on the subject had an observational design. ( Table 5 ) Recently, a recommendation from the American Academy of Sleep Medicine was published, summarizing the selection of the main medications used. 45

### Melatonin

Two double-blind randomized trials evaluated immediate-release melatonin in RBD with positive results in reducing the dream-acting frequency and REM sleep without atonia. The evaluation used the Global Clinical Impression Scale and specific RBD scales, besides PSG. 46 47 Other observational studies have also documented the effectiveness of this medication. The dosage ranged from 3 to 15 mg per day at bedtime. Higher dosages were not recorded. 45

Extended-release melatonin for RBD treatment was evaluated in two double-blind randomized trials. There was no difference in the frequency of acting-out episodes in either. 48 49 Therefore, there is no evidence for the recommendation of extended-release melatonin for RBD treatment.

In Brazil, melatonin is considered a dietary supplement and is available for sale in pharmacies at low doses. Therefore, higher dosages, such as the recommended RBD treatment, require handling the active ingredient in compounding pharmacies or importing from other countries. Its main adverse effects include headache, excessive drowsiness, nausea, and mental confusion.

### Clonazepam

Clonazepam was the first medication used in RBD patients since its description in 1986. 2 Several observational studies have documented improvements in RBD symptoms. 50 51 A single double-blind, randomized trial did not show a difference between patients who used clonazepam from those who received a placebo. However, RBD diagnosis relied on a simple question, as this study did not use PSG as a diagnostic method. 52

Clonazepam is a long-acting benzodiazepine with GABAergic action, increasing inhibition by stimulating chloride channels in the central nervous system. The doses should be low, between 0.25 and 1 mg at bedtime. Higher doses can be used if the patient tolerates it. The main side effects include excessive drowsiness, cognitive impairment, imbalance, risk of dependence, and withdrawal. Therefore, clonazepam should be prescribed with caution for elderly patients with neurodegenerative diseases. 45

Although no study has investigated this specific combination, empiric use of melatonin with clonazepam can be considered for some patients, particularly in refractory cases. The action of different mechanisms and pathways in the central nervous system may help to control dream enactment manifestations.

### Rivastigmine

Transdermal rivastigmine for RBD treatment in patients with PD and MCI has been documented in clinical trials. 53 54 Its use for isolated RBD has yet to be evaluated. Rivastigmine is an acetylcholinesterase inhibitor, leading to increased acetylcholine in the central nervous system by inhibiting the enzyme responsible for its breakdown. The dosage varies from 4.6 to 13.3 mg in a transdermal patch, with replacement every 24 hours. The main adverse effects are nausea, vomiting, anorexia, skin irritation, headache, and bradycardia. 45 53 54

### Dopaminergic agonists

Two studies showed divergent results on the effectiveness of pramipexole in RBD. An open-label study with 15 patients with isolated RBD showed improvement with doses of 0.125 to 0.375 mg/ day after a four-week trial. 55 Another study in patients with PD and RBD did not show a difference after three months of medication use. 56 A recent recommendation from AASM, from observational studies, suggests its use in patients with RBD and elevated periodic limb movement. 45

Pramipexole is a dopaminergic agonist medication used for motor symptoms of PD, restless legs syndrome (RLS), and periodic limb movement disorder. Adverse effects include nausea, headache, excessive sleepiness, impulse control disorder, and augmentation in patients with RLS.

A recent open clinical trial with rotigotine enrolling 11 patients over 20 weeks demonstrated improvement in acting behaviors and bed fall frequency but with persistent RWA. 57 Further studies are needed to confirm these findings.

### Yokukansan (Yi-Gan San)

Yokukansan (YKS) is an herbal treatment (known as Yi-Gan San in Chinese) made up of seven herbal ingredients: Japanese Angelica root, Uncaria hook, Cnidium rhizome, *Atractylodes lancea* rhizome, *Poria sclerotium* , Bupleurum root, and Glycyrrhiza. Two retrospective observational studies demonstrated an improvement in the frequency of acting out during sleep episodes. 58 59

### Safinamide

Safinamide is an α-aminoamide that has both dopaminergic and non-dopaminergic mechanisms of action, including inhibition of monoamine oxidase-B (MAO-B) sodium (Na + ) channel blockade and modulation of stimulated release of glutamate. A longitudinal, cross-over study registered a signiﬁcant reduction of RBD symptoms in PD patients by questionnaire-Hong Kong-score (RBDQ-HS), mainly for two individual RBDQ-HK-items (dream-related movements and failing out of bed), and in REM sleep atonia, evaluated by PSG. 60

### Ramelteon

Ramelteon is a melatoninergic agonist acting on MT1 and MT2 receptors. Two separate studies evaluated the effect of this medication on RBD. In one multicenter, open-label study conducted on patients with PD, there was a reduction in the frequency of RBD symptoms, as assessed by scales. On the other hand, the other open-labeled trial involving patients with isolated RBD did not show any difference in symptoms or REM sleep atonia. 61 62

### Drugs with negative effects on trials

A double-blind, randomized trial evaluating cannabidiol in 33 patients with Parkinson's disease and RBD did not show a difference between the evaluated groups, using 75 and 300 mg/ day for 14 weeks. 63

Nelotanserin, a selective 5-HT (2A) inverse agonist, was evaluated in a multicenter, double-blind, randomized trial for RBD associated with DLB (n = 33). The study showed no difference in RBD symptom control between groups. 64

5-hydroxytryptophan was evaluated in a double-blind, randomized study for RBD in PD. The study included eighteen patients with a crossover design. There was no difference in RBD symptoms frequency nor the REM sleep atonia indices. 65

## PERSPECTIVES

RBD may precede the onset of cognitive and motor symptoms related to synucleinopathies. Future studies should define clinical, radiological, laboratory, and neurophysiological variables (“biomarkers) that can be used, individually or in combination, to predict the risk of developing synucleinopathies, and the associated timelines, ideally forecasting the type of future pathology. 42 These biomarkers could potentially become the targets of neuroprotective strategies that could prevent the emergence, or slow down the progression, of neurodegenerative diseases. Currently, a clinical study registered in Clinical Trials is investigating the potential neuroprotective effect of idebenone in these patients. 66

Many studies tried to develop time-saving computerized methods for quantifying and detecting RWA. The advent of artificial intelligence, with techniques of deep learning and machine learning, has been used in recent studies with patients with RBD. 67 68 Shortly, these technologies may assist us in the polysomnography analysis (evaluation of muscular activity, subtle movements in the video, or even electroencephalographic activity), neuroimaging assessment, and screening of homemade videos.

Furthermore, RBD may change the global neurochemical balance in areas of the central nervous system broader than the specific atonia-generating brainstem circuitry, which can provide a dynamic model of interaction between the brainstem and other CNS structures. Thus, integrated with clinical and neuroimaging findings, neurophysiological studies will provide novel insights into mechanisms underlying cortical and brainstem dysfunction in RBD and probably offer additional future therapeutic opportunities.

## PROGNOSTIC COUNSELING AND ETHICAL IMPLICATIONS REGARDING IRBD LONG-TERM PROGNOSIS

Since it is now well-established by multiple studies published in the medical literature that up to 90% of patients ≥ 50 years old with iRBD will eventually develop an alpha-synucleinopathy, the important issues of prognostic counseling and ethical implications must be considered. A comprehensive review by experts on this topic (including one of the authors, CHS) has recently been published, 69 and will now be summarized. The most common ethical model for the physician-patient relationship is “shared decision-making”. In this context, disclosure of the high risk of iRBD for the future emergence of a synucleinopathy is almost always indicated. The physician should exercise appropriate sensitivity in this process, as a worrisome medical prognosis will be shared, and with no neuroprotective (“disease-modifying”) agent currently available to slow down or halt the progression from iRBD to overt synucleinopathy. Disclosure in this context will enable patients and their families to suitably prepare, in multiple ways, for the future.

Also, appropriate information should be given about the signs and symptoms of each synucleinopathy. A healthy lifestyle, including regular exercise, should be encouraged. Additionally, enrollment in longitudinal cohort studies, currently available in North America (NAPS2), 70 Italy (FARPRESTO), 71 and across other countries (International RBD Study Group) 72 can help empower patients by participating in studies to both learn more about the progression of iRBD to overt synucleinopathy and to also participate in future studies testing promising neuroprotective agents.

Furthermore, shared decision-making is a mode of communication between the physician and patient that focuses on understanding the patient's values and preferences, and then customizing the sharing of sensitive and serious information with these values in mind. In this context, the patient is given the opportunity for complete respect of their preferences as the physician attempts to understand the extent to which the patient understands the nature of their prodromal disease (i.e. iRBD) and their high risk for developing parkinsonism or dementia. Shared decision-making is a process that often requires sequential conversations during multiple clinical visits. This process can evolve naturally as information about the patient's individual case and disease course is continuously assessed and updated in the clinical setting. Importantly, a foundation of mutual trust is built gradually that will enable more detailed conversations and information sharing to take place over time.

For patients who appear excessively apprehensive or distressed at the time of iRBD diagnosis, a “watchful waiting” approach for prognostic counseling should be considered, in which the physician repeatedly assesses the patient for the best timing and the best means of delivering this critical information. This approach in this scenario may offer some advantages, such as building a deeper mutual rapport and trust with the patient over time, and serial exams may reveal important symptoms or signs signaling the patients' own individual course, enabling an appropriately tailored prognostic discussion.

Another important consideration must be acknowledged in this process, as most patients with iRBD, along with their family and friends, will likely learn about the strong neurodegenerative risk with iRBD through the internet or other widely available sources. Therefore, without prompt physician discussion on this topic, not only might learning such information on one's own as a patient be alarming, it might also undermine trust in the physician if this risk had not been previously brought up as a topic for discussion. The proposed approach favors prognostic counseling for most patients following an initial PSG-confirmed iRBD diagnosis.

## LEGAL IMPLICATIONS OF AGGRESSIVE AND VIOLENT RBD BEHAVIORS

The typical clinical profile of chronic RBD consists of a middle-aged or older man with aggressive dream-enacting behaviors that cause repeated injury to himself and/or his wife. This profile was revealed in the first two large published series on RBD, involving 96 and 93 patients, respectively. 23 73 In these two series, male predominance was 87.5% and 87%, mean age at RBD onset was 52 years and 61 years, dream-enacting behaviors were reported in 87% and 93% of patients, and sleep-related injury as the chief complaint was reported in 79% and 97% of patients. Injuries included ecchymoses, subdural hematomas, lacerations (arteries, nerves, tendons), fractures (including high cervical—C2), dislocations, and other injuries.

A review of published cases of RBD that were associated with potentially lethal behaviors identified choking/headlock in 22–24 patients, diving from bed in 10 patients, defenestration/near-defenestration in 7 patients, and punching a pregnant bed partner in 2 patients. 74 A remarkable case of repeated RBD injury involved a 63-year-old man from China whose four consecutive wives had divorced him because of his aggressive and violent dream-enacting behaviors, including repeated biting. 75 With his first wife, one night he dreamed that he was eating an apple, but instead he was biting her ear. On subsequent nights, during similar dreams, he would bite her ears, nose, and face, which culminated in his wife divorcing him after four years of marriage. His 3 next marriages were also terminated by the wives on account of his repeated RBD-related sleep violence, including aggressive biting during dreams. In addition, three brief relationships with girlfriends were also terminated for the same RBD-related reasons. Also, in a series of 203 consecutive idiopathic RBD patients, the prevalence of biting in RBD was 8.4%, which usually involved bed partners. 76 Nevertheless, a recent comprehensive review of sleep-related homicide and attempted homicide did not find any case involving RBD, only NREM parasomnias. 77

Mahowald et al. have developed the following guidelines to assist in the evaluation of forensic cases involving sleep-related violence 78 79 :

- There should be a reason to suspect a genuine sleep disorder, either based on historical evidence or formal evaluation in a sleep laboratory. Previous episodes, with either benign or harmful outcomes, should have occurred before;
- The duration of the action is typically brief, lasting only a few minutes;
- The behavior is sudden, immediate, impulsive, and appears to lack any logical motivation. It is completely inappropriate considering the overall situation and is uncharacteristic of the individual when awake. There is no evidence of premeditation;
- The person affected is usually someone who happened to be present at the time and may have triggered the arousal;
- There is no attempt to escape, hide, or cover up the actions. It is evident that the person was unaware during the event;
- There is usually some level of amnesia for the event, although it may not be complete.

Comment on Guideline 6: In the largest series on iRBD published to date on 203 patients, 40% of patients were not aware of their dream-enacting behaviors (DEB), which their spouses clearly observed as DEB. 76 The abnormal dreams were often recalled by this subgroup of RBD patients, but not the DEB.

**Table 5:** Medications used in REM sleep behavior disorder with positive trial results.

Medication	Dose	Study type	Population
Clonazepam	0.25 - 2 mg	One open, randomized trial and observational studies (2,17,18)	Isolated RBD PD
Melatonin (immediate release)	1 - 15 mg	Two double-blind, placebo-controlled, trials (47,48)	Isolated RBD; PD; Narcolepsy
Rivastigmine	4.6–13.3 mg	Two placebo-controlled, cross-over, open trial (54,55)	PD; MCI
Pramipexole	0.125 - 0.375 mg	One open-label trial (56)	Isolated RBD
Safinamide	50 mg	One double-blind, randomized trial (61)	PD
Rotigotine	2–16 mg	Prospective open-label, trial (58)	PD

Abbreviations: PD, Parkinson's disease; MCI, Mild cognitive impairment; RBD, REM sleep behavior disorder.
